# Unintended importation of tropical jumping spiders (Salticidae) into a laboratory monkey colony via banana supply

**DOI:** 10.5194/pb-7-13-2020

**Published:** 2020-09-04

**Authors:** Roland Plesker, Jürgen Berger

**Affiliations:** 1 Paul-Ehrlich-Institut, Paul-Ehrlich-Str. 51-59, 63225 Langen, Germany; 2 Max-Planck-Institut für Entwicklungsbiologie, Tübingen, Germany

## Abstract

This report describes a case of unintended importation of tropical baby jumping spiders to a laboratory monkey colony. The spiders were detected in a cocoon attached to a banana for monkey consumption. In identifying the family of spiders as jumping spiders (Salticidae), it turned out that these spiders would not have been venomous to humans and they most likely would not have had the potential to establish a new spider colony in the facility.

## 1 Introduction and literature

In a laboratory monkey colony, food is not only used as the necessary fulfilment of nutritional needs of the monkeys, but it is also an important tool in environmental enrichment (Newberry, 1995). In addition, food is used in training procedures (Westlund, 2015). Therefore, often not only pellets are offered to the monkeys, but also fruits and vegetables to make life more interesting for the animals in a laboratory setting.

These fruits and vegetables are often delivered from a central market, where fruits and vegetables are offered for human consumption. Sometimes, the covering boxes unintendedly contain unwanted domestic vermin (Plesker, 2005). In addition, on the internet as well as in newspapers and in scientific literature, reports are given on the appearance of unwanted animals – mostly spiders – in bulks of fruits (often bananas or grapes), originating from tropical countries (Nentwig, 2015; Vetter et al., 2014; Bosselaers, 2013; Nentwig and Kobelt, 2010; Van Keer, 2010, 2007; Reed, 2004). This happens although, for example, bananas are washed and disinfected before packing, cooled during transportation, and treated with ethene before they reach the markets.

In principle, every spider with venom glands must be considered as venomous, not always for humans, but also for other animals or insects (Maretic, 1987). However, only few spiders are of medical and veterinary importance (Mullen and Vetter, 2019; Vetter and Isbister, 2008; Wilson and King, 1990). Despite this fact, internet and newspaper reports often deal with the possibility that the imported individuals might be venomous to humans (e.g. https://www.nationalgeographic.com/news/2014/11/141110-spiders-bananas-fruit-food-world-toxic-animals/, last access: 31 August 2020). As a consequence of these reports, when imported spiders are detected, the fear often arises that they might be venomous to humans (Lucas and Meier, 2017; White, 2000; Maretic, 1987).

In order to raise the awareness of the problem of importing unwanted tropical spiders via food supply for persons in contact with laboratory primates, we here report a case of importation of tropical spiders (Salticidae) via bananas to our colony.

## 2 Material and methods

The monkey colony currently contains 12 African green monkeys (*Chlorocebus aethiops*), 7 pig-tailed macaques (*Macaca nemestrina*) and 2 rhesus macaques (*Macaca mulatta*) for experimental, vaccine-related research.

At the institute, the monkeys are housed in groups in an indoor area with controlled access. There is only one monkey species per room. The cages are made of steel with a basic size of 300cm×375cm×225cm (or up to triple the size). Large windows allow the monkeys to observe the outside environment. Natural branches, ropes, nets, bedding, mirrors, KONG toys, puzzle feeders, Prima-Hedrons, music and television are supplied for environmental enrichment. The housing is in accordance with European and German animal welfare legislation.

The monkeys' diet in our colony consists of monkey pellets ad libitum (Trio Munch^®^, Special Diet Services/Mazuri, Witham, England) in the morning and seasonal vegetables and fruits twice weekly in the afternoon. Some fruits (like bananas) are not peeled off before they are offered to the monkeys, in order to keep the monkeys busy with the food.

The bananas in this case were delivered from the Frankfurt central food market via a local fruit dealer. Their origin could be traced back to a food dealer in Medellín, Colombia, with the help of information given on a label of the covering carton.

When bananas reach the institute, they are routinely unpacked from the cartons, visually inspected and stored in a refrigerator until use. This happens inside the monkey unit, in a separate kitchen.

When the particular banana was detected in this case, first, pictures were taken. The banana was then placed into an air-tight glass filled with a 4 % formaldehyde solution to prevent the spread of vermin. After 24 h, the cocoon was opened and, again, pictures were taken. Individual baby spiders were then photographed and identified using a stereomicroscope (Zeiss Stemi 2000-C, Oberkochen, Germany) together with a digital camera (AxioCam Color, Oberkochen, Germany).

**Figure 1:**
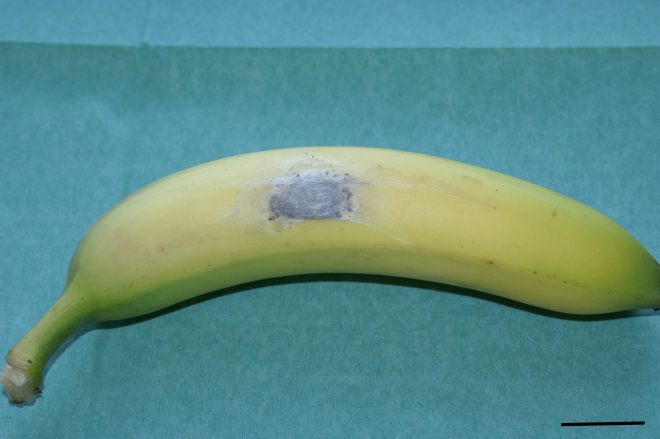
Banana delivered with a spider cocoon on the surface (scale bar = 4 cm).

After washing, the formalin-fixed spiders were dehydrated in a graduated series of ethanol followed by critical point drying with ${\mathrm{CO}}_{2}$ in a critical point dryer (Polaron E 3000, Polaron Equipment Ltd, Watford, UK). Finally, the spiders were sputter coated with a 10 nm layer of Au / Pd (Bal-Tec MED 010, Balzers Union AG, Balzers, Liechtenstein) and examined with a Hitachi S-800 field emission scanning electron microscope (Hitachi High-Tech Europe GmbH, Krefeld, Germany) at an accelerating voltage of 15 kV.

## 3 Results

When taken out of the covering carton, a flat oval beige-white cocoon (5 cm × 2.5 cm) was discovered on the surface of one banana (Fig. 1). Within the cocoon, one could adumbrate multiple small black silhouettes. Unfortunately, we could not confirm whether these silhouettes were moving or not.

**Figure 2:**
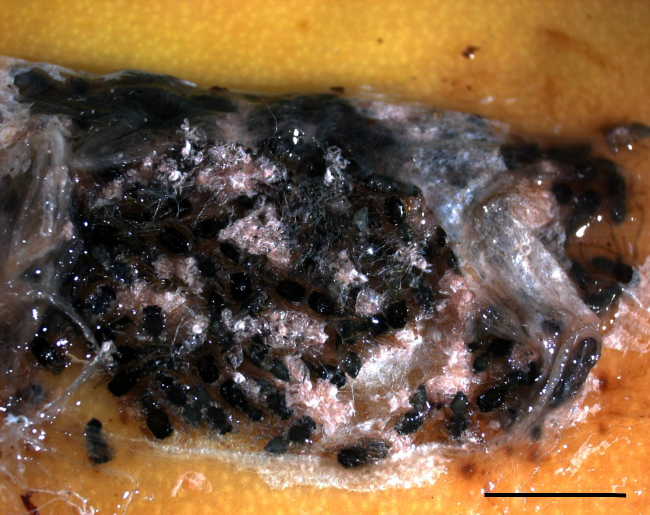
Closer view of an opened spider cocoon at the surface of a banana after formalin fixation: about 100 black individuals (scale bar = 1 cm).

**Figure 3:**
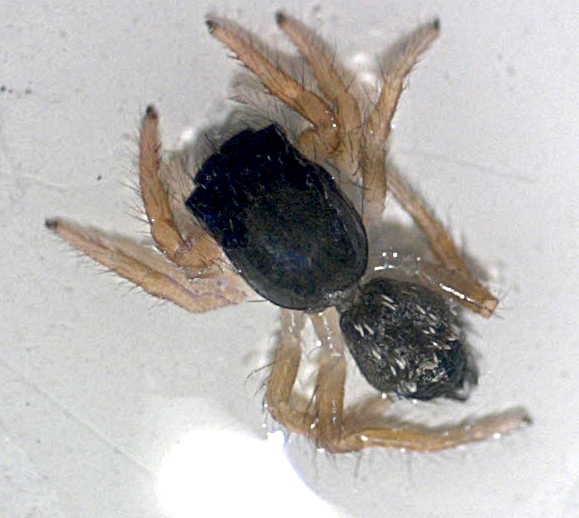
Single tropical baby spider of a cocoon on a banana surface: note the larger rectangular cephalothorax and the smaller oval abdomen (0.4 cm in total body length).

When the cocoon was opened after 24 h of formaldehyde fixation, approximately 100 well-preserved black baby spiders became visible (Fig. 2). They were up to 4 mm in body length. Their black body was divided into a larger (two-thirds) more rectangular prosoma (cephalothorax) and a smaller (one-third) oval opisthosoma (abdomen). Both were separated by a slim petiolus. In front and on the dorsal part, the prosoma contained eight eyes (four pairs) of different sizes, with the anterior median pair of eyes being the largest. Four pairs of ochre haired legs were seen in a prograde position (two legs to the front, two legs to the back). On the dorsal part of the abdomen, prominent white hairs were located in two double lines (Fig. 3). These characteristics identified the baby spiders as members of the family of jumping spiders (Salticidae). Unfortunately, since all individuals were babies, it was not possible to further determine a particular genus or species.

**Figure 4:**
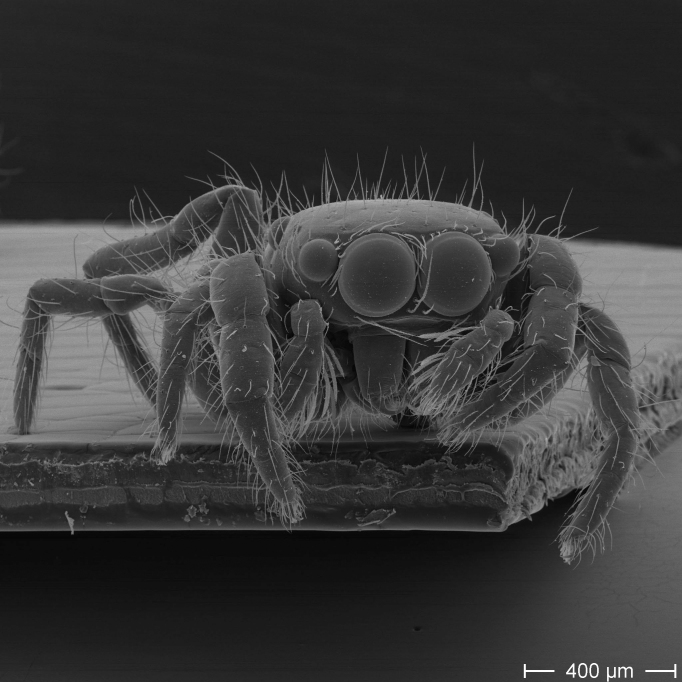
Scanning electron microscopical image of a baby spider from a cocoon on a banana: large anterior median pair of eyes indicating jumping spiders (Salticidae).

In scanning electron microscopy, the spiders displayed flat faces with a large anterior median pair of eyes (Fig. 4) and small posterior median eyes. The legs contained no spines.

## 4 Discussion

If spiders are found in boxes of imported bananas, the first impulse is to call them “banana spiders” and to assume that they are venomous to humans. However, two out of the three types of “typical” banana spiders are not dangerous: the golden silk orb weaver (*Nephila*) and the Hawaiian garden spider (*Argiope appensa)*. Only the Brazilian wandering spider (*Phoneutria nigriventer*) is venomous. However, it is obvious that the spiders presented in this case do not belong to these larger banana spiders; they belong to the family of the jumping spiders (Salticidae).

The family of jumping spiders is very large and contains currently about 600 genera and 6000 species. This means this family covers 13 % of all spiders. They can easily be identified by their rectangular cephalothorax, their large anterior median pair of eyes, their flat faces and the missing spines on the legs (Richman et al., 2005).

When the cocoon was first discovered, it was intuitively assumed that spiders might be inside. In addition, three questions for our institution were raised immediately:

1. Is there a possibility that the spiders might leave the cocoon?
2. Are these spiders venomous to humans/the staff?
3. Is there a possibility that the spiders might establish a spider colony within the animal unit?

In order to rigorously exclude any of these possibilities, the banana was deposited in formaldehyde solution and in an air-tight glass. However, due to this reflex, we were unable to find out whether the baby spiders within the cocoon were alive or already dead at the time of discovery. However, the very good preservation of their bodies might be a hint that they might still have been alive when the bananas entered the animal unit. So, probably, they would have had the potential to leave the cocoon under appropriate conditions. Since, in our case, the kitchen where the fruits and vegetables are unpacked and handled is part of the monkey unit, it is absolutely realistic to be concerned about a potential spread of imported spiders in the whole area.

When the spiders were identified as jumping baby spiders, it was clear that they would not have been venomous to humans since jumping spiders in general are not venomous to humans (Mullen and Vetter, 2019; Vetter and Isbister, 2008). In addition, it seems to be unlikely that these jumping spiders would have had the potential to establish a new spider colony within the animal unit, since – with the spiders originating from a banana plantation in Columbia – the climate in the animal unit would not be moist enough. In addition, daily cleaning in the facility would reduce both the amount of available food for the spiders and the number of places to hide. Finally, living as a spider in a monkey room would not be desirable, since monkeys have the tendency to grasp and consume anything that moves. In addition, the grooming behaviour among monkeys would increase the risk of being detected and consumed.

When a cocoon is discovered on a banana, it must be considered whether the cocoon was attached to the fruit in the banana plantation itself or whether it was secondarily attached to the banana during transportation. In principle, spiders have been detected in banana plantations (Harrison, 1968), and the first author himself has seen similar cocoons on banana bunches in Vietnam. Therefore, the attachment definitely could have happened in the banana plantation itself. On the other hand, ships as well as (more modern) containers are cleaned (and in part disinfected) on a regular basis; this makes it unlikely for the cocoon to be attached to the banana during transportation.

Since this report documents the occurrence of tropical spiders in banana cartons delivered from food markets in Germany, it stresses the following:

a. the need for a separate “arrival” room for incoming fruits and vegetables,
b. the need for the proper storage of fruits and vegetables until they are consumed,
c. consistent peeling and washing of fruits and vegetables,
d. the need for an accurate visual control of fruits and vegetables (especially bananas and grapes) before offering them to the animals.

## 5 Conclusions

This report describes a case of unintended importation of baby jumping spiders attached to a banana to a laboratory monkey colony. In identifying the family of spiders as jumping spiders (Salticidae), it turned out that these spiders would not have been venomous to humans and they most likely would not have had the potential to establish a new spider colony in the facility. This finding stresses the importance of accurate visual control and storage of vegetables and fruits reaching laboratory animal colonies.

## Data Availability

Pictures of the spiders are available via the corresponding author.
